# Retrieval of Surface Soil Moisture at Field Scale Using Sentinel-1 SAR Data

**DOI:** 10.3390/s25103065

**Published:** 2025-05-13

**Authors:** Partha Deb Roy, Subhadip Dey, Narayanarao Bhogapurapu, Somsubhra Chakraborty

**Affiliations:** 1 Agricultural and Food Engineering Department, Indian Institute of Technology Kharagpur, Kharagpur 721302, India; partha.slg09@gmail.com (P.D.R.); sdey23@agfe.iitkgp.ac.in (S.D.); 2 ICAR-Indian Institute of Water Management, Bhubaneswar 751023, India; 3 Microwave Remote Sensing Laboratory (MIRSL), University of Massachusetts Amherst, Amherst, MA 01003, USA; narayanarao.bhogapurapu@gmail.com

**Keywords:** SAR, soil moisture, vegetation, radar, volume scattering

## Abstract

The presence of vegetation in agricultural fields affects the accuracy of soil moisture retrieval using synthetic aperture radar (SAR) data. As a result, the estimation of soil moisture using the existing Oh model produces high error values. The magnitude of this error primarily depends upon the nature of crops, crop coverage, and the roughness of the field. Hence, in this study, along with the Oh model, we proposed a novel approach using model-based decomposition to reduce the volume contribution of the vegetation. This proposed method is employed on fallow as well as different crop fields in the summer of 2023 in the Kharagpur region of India using the Sentinel-1 dual polarimetric SAR data. The Root Mean Square Error (RMSE) of the proposed method is ≈25% to 52% lower over different crop types as compared to the existing Oh model. Moreover, the proposed method is also compared with the Chang model, designed to estimate soil moisture in vegetative fields. The proposed method exhibits RMSE that is around ≈10% to 17% lower across various crop kinds, in comparison to the Chang model. Thus, the proposed novel approach, with the advantage of not requiring in situ plant descriptors, will simplify the application of dual polarimetric SAR data for soil moisture estimation in a variety of land-use scenarios.

## 1. Introduction

The conventional methods of ground measurement pose significant challenges in obtaining a timely distribution of soil moisture (SM) in an extensive area and effectively recording spatial changes in SM at the field level. The use of microwave wavelength to assess SM has made significant advancements over the past few decades [1,2,3,4]. Several theoretical models, including the physical optics model (PO), the small perturbation model (SPM), and the geometrical optics model (GO), were proposed in the early days to establish the relationship between microwave backscatter and bare soil surface. However, the utilization of these models required improvement due to restrictive assumptions used in cases of different surface roughness conditions [5]. The Integral Equation Model (IEM), formulated by Fung et al. [6], which overlaps SPM and the Kirchhoff approximation to simulate backscattering coefficients for a rough surface, and its further development as an advanced IEM (AIEM) [7] to keep all the polarization correlation terms, has been inverted to estimate SM. However, the complex components of this model and the inherent correlation between soil dielectric constant and active microwave data provide challenges in directly determining SM and roughness parameters from the synthetic aperture radar (SAR) data.

Henceforth, semi-empirical inversion models with reduced parameter demands to predict SM from a wide range of surfaces using SAR data were developed. In this regard, Oh developed a semiempirical polarimetric model to derive the volumetric SM and Root Mean Square (RMS) surface height from co-polarization $\left(\sigma_{HH}^{0}/\sigma_{VV}^{0}\right)$ and cross-polarization ratios $\left(\sigma_{VH}^{0}/\sigma_{VV}^{0}\right)$ over different bare soil surfaces [8]. Several researchers applied the Oh model to invert the SM and obtained mixed results. Baghdadi et al. [9], Panciera et al. [10], Choker et al. [11], etc., reported a good simulation of backscatter by using the Oh model. However, Wang et al. [12] reported a discrepancy in the SM retrieval by the Oh model and SIR-C measurements. Wang et al. [13] also reported disagreement in soil parameters retrival by the Oh model and ground-truth data. Another popular semiempirical model to retrieve SM from co-polarized backscattering coefficients was developed by Dubois et al. [5]. However, the accuracy of these models was compromised when they encountered vegetation-covered fields.

The backscatter of SAR over an area with vegetation comprises the scattering from the earth, the vegetation, and the interaction between the two. Therefore, isolating the impacts of vegetation backscattering is imperative to enhance the model’s accuracy. Henceforth, to mitigate the vegetation effect in retrieving SM, the canopy/vegetation scattering theory was frequently utilized in several semiempirical models, such as the De Roo model [14] and Water Cloud Model (WCM) [15,16]. Various in situ measurable factors, such as vegetation descriptors, are employed to mitigate the influence of the vegetation in those approaches. However, the suitability of these models for regional-scale applications is still being investigated [17]. Other methodologies that had emerged based on other ideas include Change Detection techniques [18,19,20], Machine Learning (ML) [21,22,23], Artificial Neural Network (ANN) [24], and Target Decomposition method [25]. In the Change Detection method, Wagner et al. [26] explore the relationship between backscatter coefficient with incident and azimuth angle and its sensitivity with the vegetation and soil moisture content. Balenzano et al. [27] improve the method by taking backscatter ratios of consecutive satellite acquisitions. However, to find multi-angle and multi-frequency data, pose a major limitation to this approach.

Subsequently, understanding scattering mechanisms in polarimetric SAR data [28,29] put up the proposition of decomposing the target scattering matrix into orthogonal components [30,31,32]. Model-based polarimetric decomposition (PD), proposed by Freeman and Durden [31], tries to differentiate the elements based on differences in scattering, viz. surface, dihedral, and vegetation volume. Many other researchers have further refined this idea [33,34,35]. The PD approach was further extended to estimate SM estimation in the agriculture field by Hajnsek et al. [36] and Jagdhuber et al. [2]. The PD is widely accepted as compared to sole mathematical-based approaches because of its strong physical basis [37]. However, this approach is mostly limited to fully polarimetric data. The availability of high-resolution dual-pol SAR data obtained by the Sentinel-1 mission at a large scale, combined with its frequent revisits, has motivated researchers globally to develop an improved algorithm for SM mapping. Although dual-pol data have less polarimetric information compared to full-pol data, the former has the advantage of a larger swath and a lower volume compared to full-pol data [38].

The available SM products from passive microwave sensors, such as soil moisture ocean salinity (SMOS) and soil moisture active passive (SMAP), have limited application due to their coarse spatial resolution. Many attempts were made to downscale those products with an optical or thermal dataset. However, several environmental factors, like a cloudy sky and smoke from wildfires, have remained major constraints. Alternatively, many researchers have attempted to downscale with high-resolution Sentinel-1 SAR data [19,39,40,41] and were able to achieve almost 1 km resolution SM products through different approaches. The change detection approach introduced by [26] is modified further to develop an algorithm that works under the framework of the Sentinel-1 mission [18,42]. Bauer-Marschallinger et al. [18] developed a globally deployable SM observation data set with a 1 km spatial resolution. However, to achieve an even finer resolution SM product at the plot level, researchers have to address the challenge of a combination of SM and soil roughness along with vegetation cover that adds bias in SM estimation. Hence, the change detection algorithm is improved through dynamic vegetation correction from optically based parameters. Gao et al. [43] used the normalized difference vegetation index (NDVI) from Sentinel-2 to correct the vegetation scattering in consecutive Sentinel-1 backscattered data for SM estimation. Subsequently, Bao et al. [44] modified the WCM with optical data from Landsat-8. Ma et al. [3] integrated Sentinel-2-derived vegetation water index (VWI) with WCM. Qiu et al. [45] integrated the physical-based Advanced Integral Equation Model (AIEM) with WCM to estimate SM at field scale. Despite the benefit of utilizing the optical product, it requires cloud-free data, which adds a limit, particularly in the rainy season [46]. Other limitations of optical indices include its reduction of sensitivity under thick vegetation cover [20,47].

Recent studies [48,49,50] have explored deep learning techniques, particularly ANN inversion algorithms, for retrieving soil moisture (SM) from Sentinel-1 data. Additionally, Liu et al. [51] proposed a regression-based convolutional neural network (CNN) framework for SM estimation. However, the requirement of a large labeled dataset, poor generalization across regions, and high computational cost are some of the limitations mentioned by researchers for using deep learning networks for SM estimation. [52,53].

The physical model-based polarimetric decomposition (PD), mostly developed for full-pol data, has been used in an attempt to retrieve SM with coherent dual-pol data by Mascolo et al. [54] due to the easy availability of Sentinel-1 data. Later, Bhogapurapu et al. [20] utilized dual-pol GRD SAR data to estimate SM in crop fields. Dey et al. [55] also proposed the extended Bragg (x-Bragg) surface scattering model for dual-pol SAR data to estimate the dielectric constant of soil. Furthermore, Chang et al. [56] proposed an approach to separate soil backscatter from total backscatter using polarimetric radar vegetation index (PRVI) and then applied an inversion technique to retrieve SM based on the soil surface backscattering coefficient from dual-pol Sentinel-1 data. These PD techniques and the high spatial resolution Sentinel-1 data have prompted the adaptation of current SM estimate methods to maximize the utilization of dual-pol SAR data.

In this regard, it might be noted that the physical models, such as Oh, can accurately estimate SM over a wide range of bare soil surfaces [10,57]. However, the estimation accuracy decreases with increased above-ground biomass for different crop types. This type of overestimation mostly happens due to the high randomness in the scattered wave. Therefore, an important question may arise on the procedure of reducing the overestimation of estimated soil moisture: can an additional scheme along with the physical models overcome the existing problem to some extent or not? Hence, this manuscript proposes a hybrid approach to minimize the vegetation effect on soil backscatter by combining the dual polarimetric decomposition method and the physical models. The proposed approach utilizes a randomly oriented and uniformly distributed volume of dipoles covariance matrix to model the vegetation on the top of the soil surface, as the canopy of any crop can have branches, twigs, and sub-branches in any possible direction. Subsequently, the modified co-pol information, which, in this case, is the first element of the covariance matrix, and vegetation subtraction is utilized in the Oh model to improve the accuracy of SM estimation for vegetative fields. Thus, this proposed method offers an advantage over existing semi-empirical models, as it does not require in situ vegetation descriptors such as plant height, leaf area index (LAI), or leaf water area index (LWAI) to mitigate vegetation influence. In addition, as the method does not use optical indices to remove vegetation effect, it can be applied to invert SM in the absence of cloud-free optical data throughout the year.

## 2. Methodology

In SM retrieval, Oh [8] developed a semi-empirical approach using the existing scattering models and the database obtained from the ground-based polarimetric scatterometers and airborne SAR systems. In particular, the model was primarily validated for different bare-ground conditions. According to the Oh model, the backscatter intensity for the vertically transmitted, horizontally received polarization ($\sigma_{\mathrm{vh}}^{0}$) can be written as

(1) $$\begin{array}{c} \sigma_{\mathrm{vh}}^{0}=0.11\;M_{v}^{0.7}{\left(\cos\theta \right)}^{2.2}\left[1-\exp\left(-0.32{\left(ks\right)}^{1.8}\right)\right] \end{array}$$

In the above, $M_{v}$ is the volumetric soil moisture in $m^{3}\;m^{-3}$, θ is the incidence angle, *k* is wave number, and *s* is the roughness height. Further, the co-pol backscatter intensity in vertically transmitted, vertically received polarization ($\sigma_{\mathrm{vv}}^{0}$) can be related as

(2) $$\begin{array}{c} \frac{\sigma_{\mathrm{vh}}^{0}}{\sigma_{\mathrm{vv}}^{0}}=0.095{\left(0.13+\sin1.5\theta \right)}^{1.4}\left(1-\exp\left[-1.3{\left(ks\right)}^{0.9}\right]\right) \end{array}$$

Now, by substituting $\sigma_{\mathrm{VH}}^{0}$ from Equations (1) and (2), the surface SM, $M_{v}$ can be rewritten as,

(3) $$\begin{array}{c} M_{v}={\left[\frac{\sigma_{\mathrm{vv}}^{0}0.095{\left(0.13+\sin1.5\theta \right)}^{1.4}\left(1-\exp\left[-1.3{\left(ks\right)}^{0.9}\right]\right)}{0.11{\left(\cos\theta \right)}^{2.2}\left[1-\exp\left(-0.32{\left(ks\right)}^{1.8}\right)\right]}\right]}^{\left(10/7\right)} \end{array}$$

According to the definition, the model is valid over soil conditions $0.04\;m^{3}\;m^{-3}<M_{v}<0.291\;m^{3}\;m^{-3}$ and 0.13<ks<6.98, 10°<θ<70°.

Although these conditions hold well for the top-soil layer during cultivation, the aboveground vegetation acts as an additional noise to backscatter intensity from the soil surface. The dual-pol coherency matrix $C_{2}$ thus has both volume and surface scattering components. As a result, this situation violates the necessary conditions of the Oh model. We used a model-based polarimetric decomposition here and used a random dipole cloud as a volume model for the dual-pol data. As a result, we separate the soil surface scattering from the total backscatter. Finally, we use the soil backscatter value for inversion in the Oh model. Therefore, in this study, we reduce the vegetation effect to enhance the estimation accuracy of SM using dual-pol SAR data.

For dual-pol SAR data, the second-order scattering information can be written in terms of a 2×2 covariance matrix:

(4) $$\begin{array}{c} C=\left[\begin{array}{cc} C_{11} & C_{12}\\ C_{21} & C_{22} \end{array}\right] \end{array}$$

The diagonal elements of C, i.e., $C_{11}$ and $C_{22}$, can be related to $\sigma_{\mathrm{VV}}^{0}$ and $\sigma_{\mathrm{VH}}^{0}$, respectively. However, the covariance information, C, may consist of a significant vegetation effect. Hence, we have removed the vegetation effect using a model-based decomposition technique [54].

We first formulate the 4×1 Stokes vector, $\vec{S}$ to apply the decomposition. The four elements of $\vec{S}$, $g_{\nu }$, can be derived from $g_{\nu }=\mathrm{tr}\left[C\sigma^{\nu }\right]$. Here, $\sigma^{\nu }$ are the Pauli basis matrices, and ‘tr’ represents the trace of a matrix. ν ranges from 0–4. In this case, $g_{0}$ is the total power of the scattered wave, $g_{1}$ corresponds to the linear horizontally or vertically polarized power, $g_{2}$ is the power at + 45° or + 135° linear polarized components, and $g_{3}$ is power at the left-hand or right-hand circular polarized component of the scattered wave.

The dual-pol Stokes vector for a random volume scatterer can be mapped as shown in Equation (5).

(5) $$\begin{array}{c} {\vec{S}}_{v}=\frac{m_{v}}{F_{p}+2}\left[\begin{array}{c} F_{p}+2\\ \pm F_{p}\\ 0\\ 0 \end{array}\right] \end{array}$$

where $m_{v}$ is the volume proportionality constant and $F_{p}$ is the single particle shape parameter. The range of $F_{p}$ varies from 0 to ∞, where 0 corresponds to the scattering from a random dihedral particle and ∞ corresponds to the scattering from a sphere. In our particular case, we found that the improvement of RMSE becomes insignificant beyond $F_{p}=2$. Hence, we set the value of $F_{p}=2$, which gives the expression of ${\vec{S}}_{v}$ as

(6) $$\begin{array}{c} {\vec{S}}_{v}=m_{v}\left[\begin{array}{c} 1\\ \pm 0.5\\ 0\\ 0 \end{array}\right] \end{array}$$

Therefore, the expansion of $\vec{S}$ can be written as

(7) $$\begin{array}{c} \vec{S}=m_{v}\;{\vec{S}}_{v}+m_{s}\;{\vec{S}}_{s} \end{array}$$

where $m_{s}$ is the proportionality constant of the scattering from the soil surface component and ${\vec{S}}_{s}$ is the Stokes vector representing the soil surface component of the wave. Key to finding a unique solution is to use the fact that ${\vec{S}}_{s}$ is always polarized. Thus,

(8) $$\begin{array}{c} \det(C2_{p})=0 \end{array}$$

(9) $$\begin{array}{c} {\vec{S}}_{s}^{T}G{\vec{S}}_{s}=0 \end{array}$$

where G is a 4×4 matrix:

$$G=\left(\begin{array}{cccc} 1 & 0 & 0 & 0\\ 0 & -1 & 0 & 0\\ 0 & 0 & -1 & 0\\ 0 & 0 & 0 & -1 \end{array}\right)$$

(10) $$\begin{array}{c} {\left(\vec{S}-m_{v}{\vec{S}}_{v}\right)}^{T}G\left(\vec{S}-m_{v}{\vec{S}}_{v}\right)=0 \end{array}$$

for the special case of a dipole cloud,

(11) $$\begin{array}{c} am_{v}^{2}+bm_{v}+c=0 \end{array}$$

$$\left\{\begin{array}{c} a={\vec{S}}_{v}^{T}G{\vec{S}}_{v}=0.75\\ b=-2{\vec{S}}_{v}^{T}G{\vec{S}}_{v}=-2s_{1}\pm 0.5s_{2}\\ c={\vec{S}}^{T}G\vec{S}=s_{1}^{2}-s_{2}^{2}-s_{3}^{2}-s_{4}^{2} \end{array}\right.$$

Only one root of this quadratic will satisfy energy conservation $m_{v}\leq s_{1}$, providing a unique solution for the volume power mv. The polarized component can now be found by simple subtraction.

Therefore, the Stokes vector of the soil surface component can be written as

(12) $$\begin{array}{c} m_{s}\;{\vec{S}}_{s}=\vec{S}-m_{v}\;{\vec{S}}_{v} \end{array}$$

Utilizing the polarized Stokes vector, we can formulate a new 2×2 covariance matrix $C^{s}$. However, in this case, the rank of $C^{s}$ becomes 1, which means that the $C_{22}$ cross-pol component of $C^{s}$ becomes 0. The modified $C_{11}$ element, i.e., $C_{11}^{s}$, of $C^{s}$ is used in Equation (3) to retrieve the surface SM.

Further, we compute the model proposed by Chang et al. [56] for comparison. However, the polarimetric radar vegetation index (PRVI)-based biomass estimation introduced by Chang et al. [58], particularly for arid and semi-arid environments, was not able to estimate the biomass in our field conditions. Therefore, we estimate biomass using the NDVI, as presented by Filella et al. [59]. Furthermore, we separated the soil backscattering coefficient from the total biomass and subsequently inverted it to estimate the SM.

## 3. Study Area and Dataset

**Figure 1:**
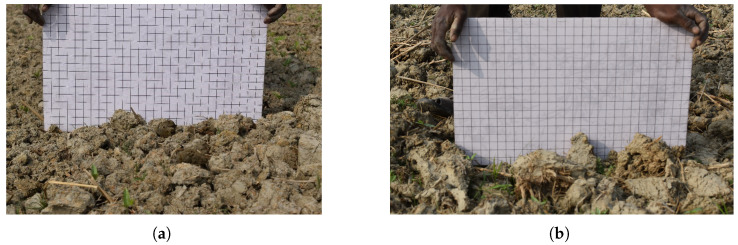
Soil roughness estimation using a roughness board in different field conditions: (**a**) and (**b**) illustrate variations in soil surface roughness across two distinct fields.

Surface soil samples (0 cm to 5 cm) were obtained with keen boxes from the farmer fields located in the Paschim Medinipur area of West Bengal. The gravimetric method was used to find out SM in the collected soil. Further, the in situ soil bulk density (BD) was determined with undisturbed soil cores (by means of a core sampler of 5 cm×5 cm length and inner diameter, respectively) from each of the fields in triplicate [60]. Weighted cores are covered with a plastic sheet to prevent moisture loss in the field and transferred to the laboratory; at 105 °C, they were oven-dried to obtain the dry weight. The volumetric SM content was then obtained by multiplying the gravimetric SM content with the respective soil BD. Moreover, soil roughness information was obtained with the help of a roughness board, and the rms height (cm) is calculated from three replications from each field (Figure 1).

**Figure 2:**
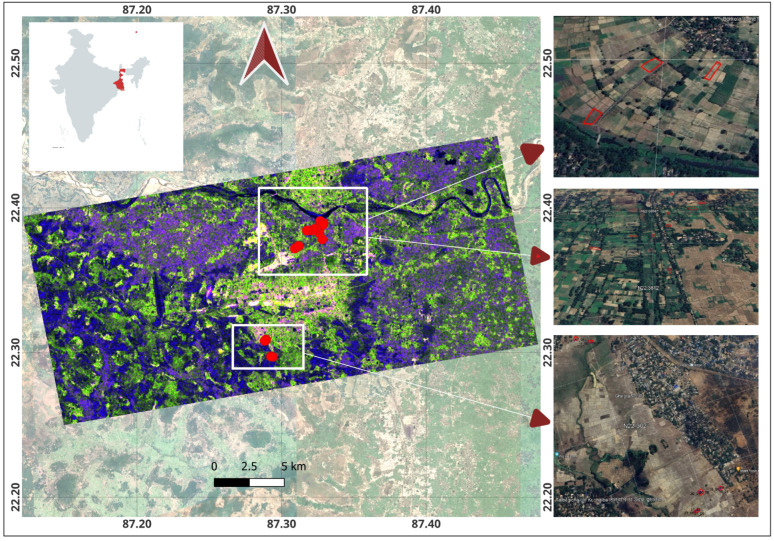
Sentinel-1 false-color composite image of study area on 3 April 2023. The areas within the two white boxes depict the two study sites from which the in situ data were collected, shown as red dots. On the right side, the Google Earth images show the general land cover characteristics over the study areas.

**Figure 3:**
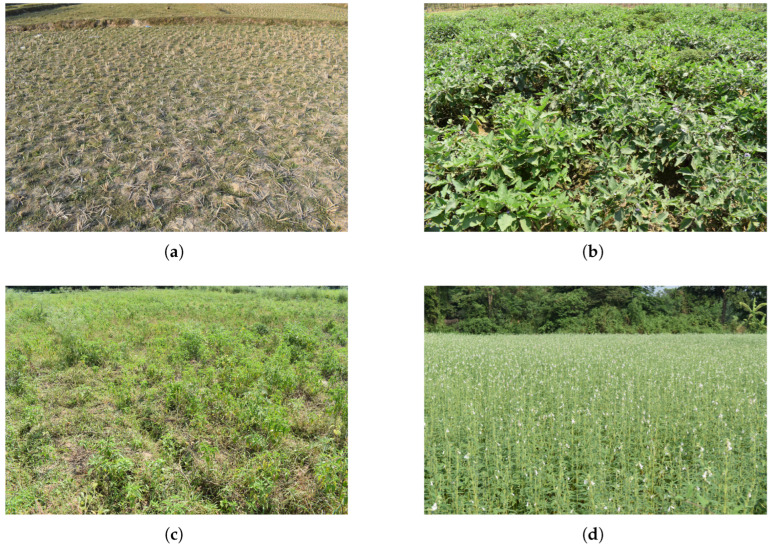
In situ images of different land cover types: (**a**) Fallow, (**b**) Brinjal, (**c**) Weed, (**d**) Sesame, selected for the study on 15 April 2023.

The test sites are spatially separated into two distinct clusters inside the Paschim Medinipur district (Figure 2). The first cluster consists of cultivated fields for vegetables and sesame, located near Barkola and neighboring villages along the Kangsabati River. The second cluster comprises rice fallow fields situated in Talbagicha, Kharagpur. These fields span a latitude range of 22.29° N to 22.40° N and a longitude range of 87.27° E to 87.35° E. The major soil type in rice fallow land in Paschim Medinipur is Paleustalfs, whereas the vegetables and sesame fields near Kangsabati river are classified as Haplaquepts [61,62]. During summer 2023, particularly from March to May, we collected a total of 514 samples, comprising four major land uses, viz. Brinjal, sesame, weed, and fallow (Figure 3). The maximum temperature reaches as high as 42 °C, while the minimum temperature varies around 25 °C in the month of April.

For this study, ten dual-pol single-look complex SAR datapoints from Sentinel-1 were chosen. We collected soil samples from the field for SM determination in synchronization with the Sentinel-1 overpass. The despeckling of the data was carried out using a 7×7 refined Lee polarimetric speckle filter [63]. Alongside this, a map of the local incident angle was prepared. The details of the Sentinel-1 data used in this study are tabulated in Table 1.

## 4. Results and Discussion

**Figure 4:**
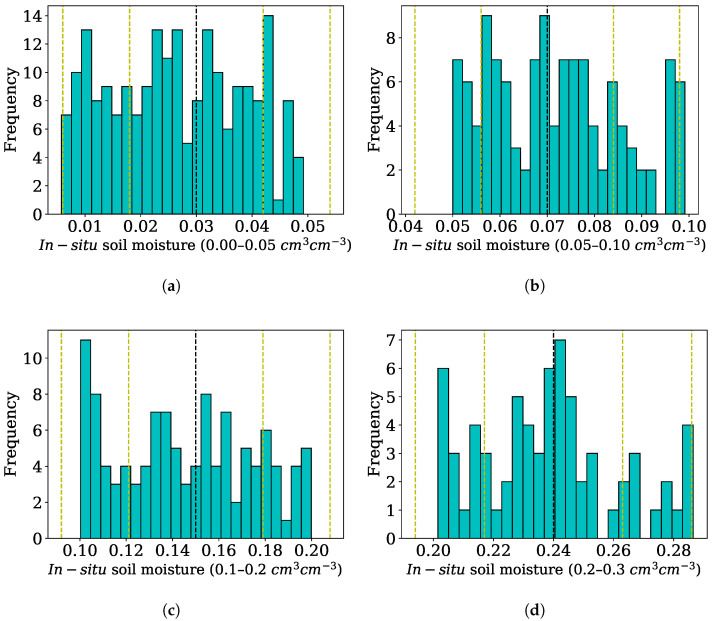
Soil moisture distribution in very dry (**a**), dry (**b**), intermediate (**c**), and wet (**d**) categories. The black dashed line at the center represents mean value and the yellow dashed lines represent 1× standard deviation and 2× standard deviations from the center.

In this study, we propose modifying the Oh method to accurately estimate the surface SM in various crop field conditions. In this context, we have analyzed the SAR backscatter data within the range of variability in the field in Table 2. Further, we categorized the entire set of samples into four SM categories: very dry (0–4.9%), dry (5–9.9%), intermediate (10–19.9%), and wet (20–29.9%), as variation in SM levels will influence the SAR backscatter. The mean soil moisture contents were found to be 3, 7, 15 and 24% in the very dry, dry, intermediate, and wet categories, respectively (Figure 4). Further, 211, 121, 113, and 69 samples were distributed, respectively, in four SM categories: very dry (0–4.9%), dry (5–9.9%), intermediate (10–19.9%), and wet (20–29.9%).

**Figure 5:**
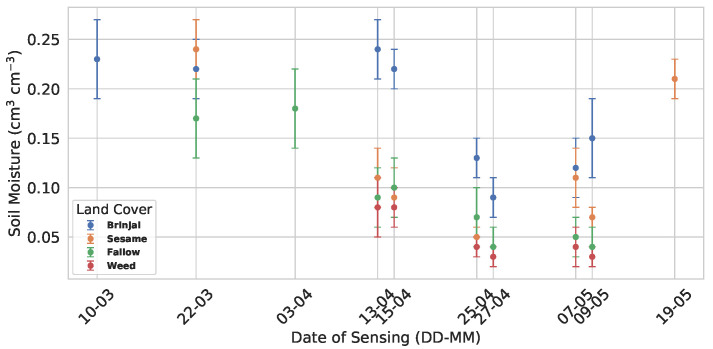
Summary of soil moisture (mean ± standard deviation) across land cover types by date of sensing (DoS).

Soil moisture (SM) varied across land cover types and sensing dates (Figure 5). Both Brinjal and sesame fields initially showed high SM (0.23 ± 0.04 cm^3^ cm^−3^ and 0.24 ± 0.03 cm^3^ cm^−3^, respectively), while fallow areas exhibited moderate values. A declining trend was observed across all land covers in April. Weed-covered plots consistently showed lower SM. Overall, clear temporal variations in SM were evident across different land cover types.

Table 2 provides the elements of the covariance matrix in the form of mean ± standard deviation values for distinct SM categories. The $C_{11}$ increases with SM content. This phenomenon might be due to the swelling impact, which was also addressed by Lu and Meyer [64] for an SM range of 5% to 20%. Alternatively, the $C_{22}$ exhibits an increase in its composition of over 5% SM, which might be associated with the soil surface roughness [8]. However, it exhibited reduced sensitivity to variations in SM content beyond that point.

**Figure 6:**
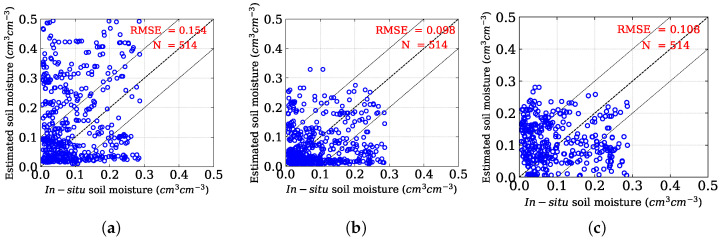
Scatterplots showing the relationship between in situ soil moisture and predicted soil moisture using (**a**) the Oh model, (**b**) the proposed method, and (**c**) the Chang method.

The performance of the proposed method is assessed statistically using the Root Mean Square Error (RMSE) values and compared with the original Oh model. Figure 6 illustrates the overall correlation between in situ SM and predicted SM obtained from both techniques. The RMSE value obtained using the Oh model is 0.154 c$m^{3}$ c$m^{-3}$, whereas the proposed approach reduces the RMSE by ≈36% as compared to the existing Oh model and yields a value of 0.098 c$m^{3}$ c$m^{-3}$, based on 514 sample points. Further, the inversion rate in the Oh method is ≈71%, whereas, in the proposed method, it is ≈89%. Therefore, the proposed model efficiently reduces the overestimation of the Oh model by removing the vegetation effect in the SAR backscatter using the volume matrix of the randomly oriented dipoles.

**Figure 7:**
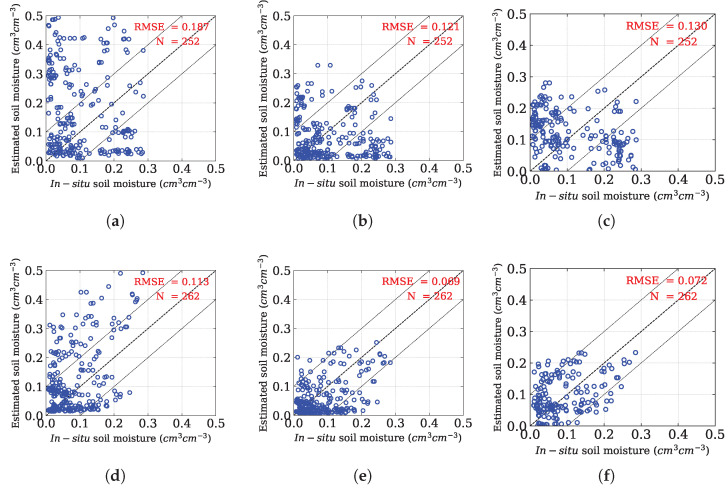
Scatterplots between in situ soil moisture and predicted soil moisture for ascending (**a**–**c**) and descending (**d**–**f**) passes, using (**a**,**d**) the Oh model, (**b**,**e**) the proposed method, and (**c**,**f**) the Chang method.

The proposed model is now independently analyzed for both ascending and descending passes. The results revealed a better soil moisture estimation as compared to the Oh method as well as the Chang model in both ascending as well as descending passes (Figure 7). However, in descending pass, the proposed method retrieves soil moisture with better accuracy (RMSE: $0.069\;{\mathrm{cm}}^{3}\;{\mathrm{cm}}^{-3}$) as compared to the ascending pass (RMSE: $0.121\;{\mathrm{cm}}^{3}\;{\mathrm{cm}}^{-3}$). Bai et al. [65] also reported improved soil moisture retrieval performance for Sentinel-1A during descending passes.

**Figure 8:**
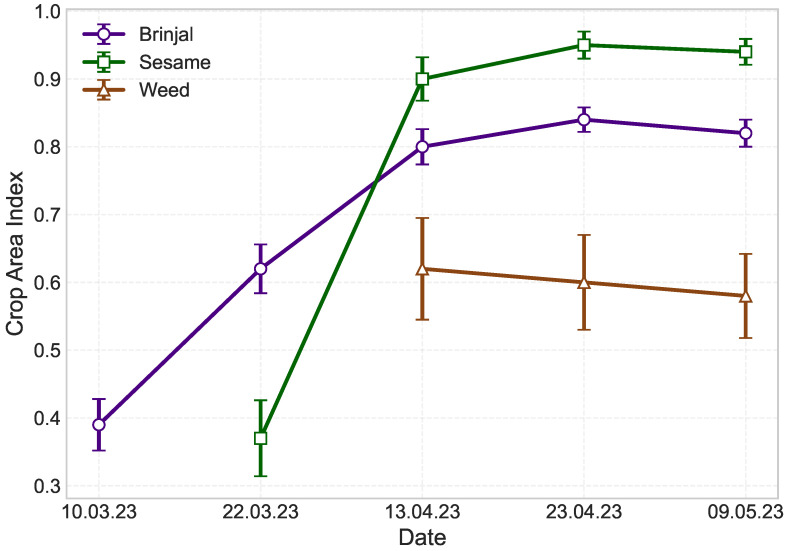
Temporal variation of Crop Area Index (CAI) for Brinjal, sesame, and weed. CAI was calculated as the ratio of crop pixels to the total number of pixels in each photograph. The error bars represent standard deviation (SD) derived from multiple images for each date.

For croplands, we have assessed the temporal dynamics through Crop Area Index (CAI) for three distinct land-use types—Brinjal, sesame, and weed, over a series of observation dates, spanning March to May (Figure 8). The analysis revealed a progressive increase in CAI for both Brinjal and sesame, indicative of steady canopy development throughout the growing period. Brinjal exhibited a gradual rise in CAI, increasing from 0.39 on 10 March to a maximum of 0.84 by 23 April. In contrast, sesame displayed a relatively sharper increase, with CAI values rising from 0.37 on 22 March to 0.95 on 23 April, suggesting rapid ground coverage once establishment was achieved. The CAI for Weed cover remained relatively stable, fluctuating slightly between 0.59 and 0.62 from mid-April to early May, indicating minimal variation during this period. The standard deviation associated with each observation indicated moderate spatial heterogeneity, particularly for Sesame during its early growth phase — a variability likely attributed to the broadcast sowing method, which naturally introduces uneven plant distribution. As the growth progress, this variability minimized, highlighting the stabilizing effect of full vegetation cover. However, the higher standard deviation for weed suggests an uneven spatial distribution within the field.

**Figure 9:**
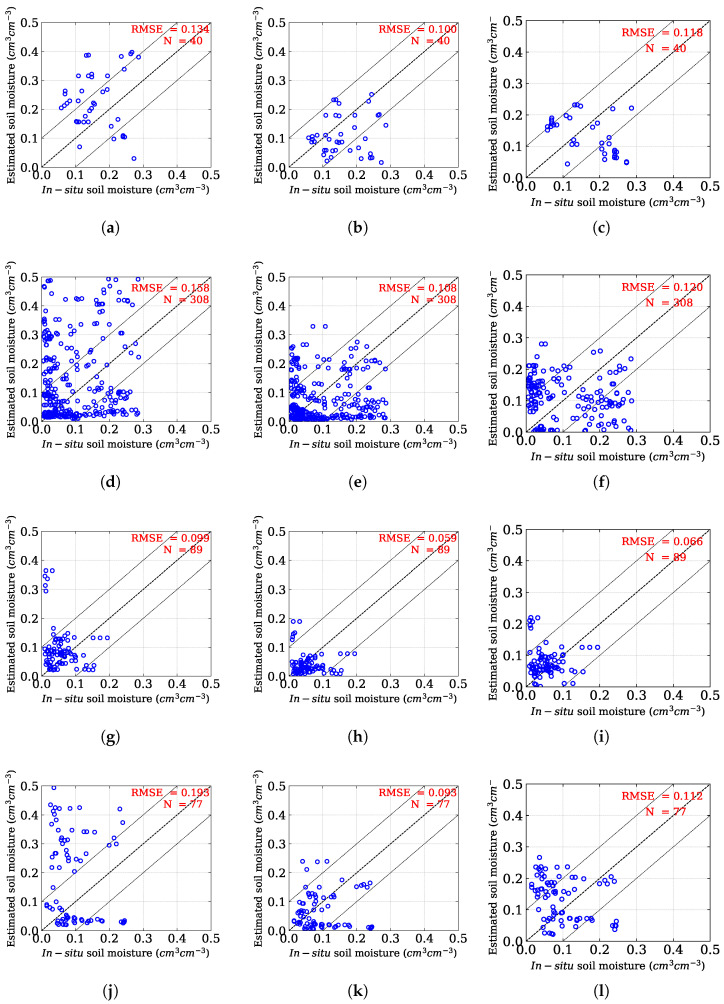
Scatterplots between in situ soil moisture and predicted soil moisture using the Oh model (**a**,**d**,**g**,**j**), the proposed method (**b**,**e**,**h**,**k**), and the Chang method (**c**,**f**,**i**,**l**) across different crop types: (**a**–**c**) Brinjal, (**d**–**f**) Fallow, (**g**–**i**) Weed, and (**j**–**l**) Sesame.

Following this, we have shown the SM retrieval efficiencies for different crop scenarios. We mainly collected data from Brinjal, sesame, weed and fallow land. Figure 9 shows the correlation plots illustrating the in situ SM and estimated SM for both methods. For Brinjal (*Solanum melongena*) plants, altogether, 40 sample points were considered for model evaluation. High error in SM estimation with the Oh model is observed with an RMSE of $0.134\;{\mathrm{cm}}^{3}\;{\mathrm{cm}}^{-3}$, as can be seen in Figure 9a. Nevertheless, the proposed technique is capable of providing a more precise estimation of SM, with an RMSE of $0.100\;{\mathrm{cm}}^{3}\;{\mathrm{cm}}^{-3}$, as shown in Figure 9b. Similarly, using both techniques, Figure 9d,e displays the SM estimation under fallow conditions. The RMSE value over fallow fields is $0.158\;{\mathrm{cm}}^{3}\;{\mathrm{cm}}^{-3}$. However, the proposed method provides a more precise estimate with an RMSE of $0.108\;{\mathrm{cm}}^{3}\;{\mathrm{cm}}^{-3}$. In the case of weed-covered field, the proposed method also performs better than the Oh method (Figure 9g,h). Sesame (*Sesamum indicum*), mostly grown as a broadcasted crop, differs from vegetables in height and ground coverage. A total of 77 sample points from these fields were collected for the study. A poor SM estimation with the Oh method is evident, with an RMSE of $0.193\;{\mathrm{cm}}^{3}\;{\mathrm{cm}}^{-3}$, as shown in Figure 9j. This phenomenon essentially shows the severe effect of vegetation during SM estimation using the Oh model. In contrast, the proposed approach yields significantly better SM estimation, with a ≈52% reduction in RMSE, as can be seen in Figure 9k. Bias score is also computed for Oh and the proposed method for different land uses, namely Brinjal, weed, fallow, and sesame, as well as the overall bias across all classes. The results indicate that the Oh model exhibits minor overestimations across all classes, ranging from 0.04 to 0.09. Specifically, the Brinjal class shows the highest bias (0.07) under Oh, whereas weed reports the lowest (0.04). Conversely, the proposed approach demonstrates reduced bias values, with all classes showing slight underestimation, ranging from −0.02 to −0.05. The overall bias was reduced by 60%, clearly demonstrating the superiority of the proposed approach as compared to the Oh model in enhancing model performance across different land use.

Further, the performance of the proposed approach was also evaluated across different phenological stages or varying plant heights of the crop. In sesame, at the seedling stage, when the plant height varied between 10 and 20 cm, the Root Mean Square Error (RMSE) was observed to be 0.15 c$m^{3}$ c$m^{-3}$. During the vegetative growth stage, with plant height ranging from 50 to 60 cm, the RMSE decreased significantly to 0.064 c$m^{3}$ c$m^{-3}$. Furthermore, at the flowering and pod development stages, when the plant height was between 100 and 120 cm, the RMSE was recorded as 0.070 c$m^{3}$ c$m^{-3}$. These results indicate that the proposed model achieved the highest accuracy during the vegetative growth stage (50–60 cm), followed by the flowering and pod development stages (100–120 cm), while comparatively higher errors were observed at the early seedling stage. In the case of Brinjal, the proposed method achieved an RMSE of 0.14 c$m^{3}$ c$m^{-3}$ when the average plant height was around 50 cm, and an RMSE of 0.13 c$m^{3}$ c$m^{-3}$, with an average plant height of around 75 to 80 cm.

We also compared our proposed model with the model-based inversion models, specifically those proposed by Chang et al. [56], for soil moisture retrieval from radar backscatter of vegetation fields. The Chang model’s RMSE for all of the samples is $0.108\;{\mathrm{cm}}^{3}\;{\mathrm{cm}}^{-3}$ in contrast to $0.098\;{\mathrm{cm}}^{3}\;{\mathrm{cm}}^{-3}$ in our proposed model (Figure 6). We also observed the Chang model for SM estimation in both fallow and individual crops to ensure a better comparison with our proposed method. We obtained an RMSE of $0.120\;{\mathrm{cm}}^{3}\;{\mathrm{cm}}^{-3}$ for 308 sample points in the fallow condition for the Chang model (Figure 9f). In the land cover of Brinjal, sesame, and weed, the Chang model recorded RMSEs of 0.118, 0.112, and $0.066\;{\mathrm{cm}}^{3}\;{\mathrm{cm}}^{-3}$, in contrast to 0.100, 0.093, and $0.059\;{\mathrm{cm}}^{3}\;{\mathrm{cm}}^{-3}$, respectively, as obtained by our proposed approach (Figure 9).

Apart from RMSE, other statistical measures or metrics, viz. the Mean Absolute Error (MAE), Mean Absolute Percentage Error (MAPE), Reduced Chi-Squared statistic ($\chi_{\nu }^{2}$) and *p*-value (*p*), are also calculated to evaluate the SM estimation by different models used in the study (Table 3). A small *p* (<0.05) in most land use indicates a significant difference among model estimations. Therefore, other effect size measures (RMSE, MAE, MAPE) are computed for evaluation. The MAE, or average absolute error, is observed in the following sequence: as-modified Oh method < Chang model < Oh model for all the crops. The MAE for the proposed method is observed at 0.07 for all the points, much lower compared to 0.09 and 0.12 in the Chang and Oh models, respectively. The MAPE, which expresses the average error as a percentage, is considerably higher in the Oh model, 3.40, followed by Chang with 2.42, and the least value of 1.70 is observed in the proposed method for all the points. The lower value of MAPE indicates the better performance of the proposed method in different land use scenarios. $\chi_{\nu }^{2}$ is another statistic that helps to compare different model performances. Here, the $\chi_{\nu }^{2}$ of the proposed method is closest to one in all the land-use scenarios comparing the other two models, which demonstrates the better estimation in the proposed method. Hence, our proposed method performed better in SM retrieval in different land use–land cover scenarios as compared to the Chang model and the Oh model. In addition, the proposed approach offers an advantage in various field scenarios due to the computational complexity associated with biomass estimation, the fitting of nonlinear equations to determine the real part of the dielectric constant, and the subsequent retrieval of soil moisture in the Chang model.

Finally, the performance of our proposed soil moisture retrieval method was compared against existing satellite-based products, including SMAP (ascending and descending pass) and SMOS. The proposed method outperformed all other products, achieving the lowest RMSE (0.098), MAE (0.072), and bias (−0.02), indicating both higher accuracy and minimal systematic deviation. In contrast, SMAP-AM (descending pass) and SMAP-PM (ascending pass) exhibited higher RMSE values of 0.139 and 0.160, respectively, with considerable positive biases (0.12 and 0.14, respectively), suggesting a consistent overestimation. Similarly, SMOS showed an RMSE of 0.129 and a BIAS of 0.11. These results demonstrate the superior reliability of the proposed method in capturing soil moisture dynamics compared to current satellite products.

**Figure 10:**
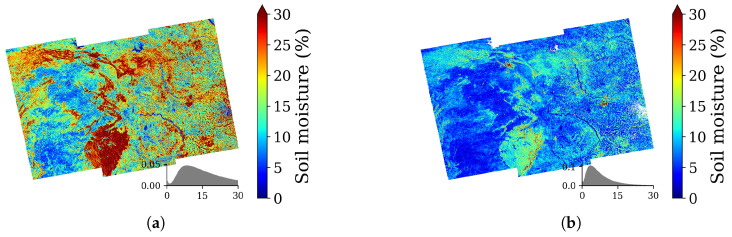
Soil moisture maps for 3 April 2023, over the study area using (**a**) Oh model and (**b**) Proposed method. Estimated soil moisture in the presence of vegetation shows a significant overestimation in (**a**).

In addition to the quantitative accuracy assessment, a soil moisture map was generated for a section of the study area to examine spatial distribution. Figure 10 presents high-resolution soil moisture maps (pixel size ≈ 15 m) derived from a Sentinel-1 image acquired on 3 April 2023, using both the Oh model and the proposed method. The selected region includes cultivated fields—such as vegetables and paddy—as well as fallow land and perennial tall vegetation along embankments. The Oh model estimates notably higher soil moisture in the northern, southern, and northwestern parts of the area, primarily over crop fields, where volume scattering affects the measurements. In contrast, the proposed method provides estimates more consistent with actual in situ soil moisture across most pixels. Thus, it effectively mitigates the overestimation caused by volume scattering in many cases. However, some red patches in the proposed method’s output—corresponding to dense vegetation or man-made structures—highlight areas where residual error remains.

Overall, the proposed model shows considerable variation over different land uses. The volume scattering component observed over Brinjal-, sesame-, and weed-covered fields is assumed to originate from a cloud of randomly oriented dielectric scatterers, consistent with the random dipole cloud model [66]. Given the heterogeneous and irregular canopy (natural randomness in orientation of stems and leaves) of crops, the random dipole cloud model provides a physically meaningful representation of the volume scattering mechanism for these vegetation classes. However, the applicability of the random dipole cloud model exhibits varying degrees of suitability, depending on the crop type. Brinjal, cultivated in the broad bed furrow method with ≈60–80 cm spacing and maximum height of around 75–80 cm at maturity, shows better SM estimation in the classical Oh method as compared to the broadcasted sesame field with tall height (up to 120 cm measured at maturity) and more dense ground coverage, as expected. Our proposed method reduces the overestimation by different magnitudes, thus reducing the errors in diverse field situations. Among different crop fields, significant improvements of 52% and 40% in RMSE are observed in the sesame- and weed-covered field, respectively. Although sesame plants exhibit an upright growth habit, their thin stems and irregularly oriented leaves introduce sufficient structural randomness within the radar resolution cell to approximate a random dipole scatterer. Moreover, the presence of heterogeneous weed flora and dried stalks in weed-covered fields, exhibiting a low average plant height ( 30 cm), can also be considered a suitable approximation for a random dipole scattering medium. We observed a 25% reduction in RMSE in Brinjal fields using the proposed approach. However, woody stems and broad leaves combined with a moderate CAI may contribute additional scattering components, along with volume scattering, limiting RMSE reduction in Brinjal fields [67]. Overall, the results demonstrates the effectiveness of the proposed method in enhancing SM estimation in various crop-covered fields, compared to the original Oh method.

Although the proposed approach significantly improves SM retrieval compared to the Oh and Chang methods, the wide range of soil moisture and the heterogeneous field situation may impose limitations on this approach. Furthermore, the stabilized backscatter response at higher soil moisture levels, combined with the inclusion of ascending orbit data, may introduce additional error in soil moisture retrieval using the proposed approach. Moreover, subsequent research aimed at implementing the proposed methodology across diverse sites with varying soil roughness and land use characteristics may facilitate the better utilization of Sentinel-1 data. It may also be utilized for longer wavelength L-band data in forthcoming missions, providing an effective solution to the issues associated with C-band data.

## 5. Conclusions

This paper proposed a method to estimate soil moisture (SM) using dual-pol synthetic aperture radar (SAR) by minimizing the effect of vegetation cover. One of the major advantages of the proposed approach is that it does not require any ancillary or in situ plant descriptors while estimating SM. The proposed method consists of a hybrid approach of coupled model-based decomposition and Oh model to derive the SM content using dual-pol SAR data. The performance of the proposed approach is extensively evaluated using Sentinel-1 C-band SAR and in situ SM data acquired over a test site in Kharagpur, India, for vegetable and oilseed crops. We obtained an overall improvement of 36% (${\mathrm{cm}}^{3}$ ${\mathrm{cm}}^{-3}$) in Root Mean Square Error with the proposed method compared to the Oh model. Specifically, the proposed method performed better in the dry to intermediate SM range by significantly reducing the overestimation. In addition, we conducted a comparison between our method and the Chang model, which is specifically developed to estimate soil moisture in vegetative fields. The proposed method resulted in a 9% (${\mathrm{cm}}^{3}$ ${\mathrm{cm}}^{-3}$) decrease in Root Mean Square Error compared to the Chang model, for all the samples. However, there is a noticeable underestimation of SM for the wet soils (>0.2 ${\mathrm{cm}}^{3}$ ${\mathrm{cm}}^{-3}$). Nevertheless, the proposed approach has potential operational scalability for SM estimations solely using dual-pol SAR data. Further, in this era of open-access SAR datasets, the simplicity and robustness of the proposed approach of SM estimation in diverse land-use settings would be a great advantage. In summary, the proposed strategy provides an advantage over alternative methods by eliminating the need for in situ plant descriptors, full polarimetric synthetic aperture radar data, or cloud-free optical data for soil moisture retrieval. Furthermore, one could readily use the approach with longer-wavelength L-band data, such as the planned for NISAR and ROSE-L missions, in the case of dense vegetation to overcome the limitations of the C-band.

## Figures and Tables

**Table 1:** Specification for Sentinel-1 data acquired for the test site.

Acquisition Date	Beam Mode	Incidence Angle Range (Deg.)	Orbit	rg (m) × az (m)
10 March 2023	IW	42.2–46.1	Ascending	13 × 14
22 March 2023	IW	41.6–46.0	Ascending	13 × 14
03 April 2023	IW	41.9–46.1	Ascending	13 × 14
13 April 2023	IW	38.1–42.2	Descending	13 × 14
15 April 2023	IW	41.9–46.1	Ascending	13 × 14
25 April 2023	IW	38.1–42.3	Descending	13 × 14
27 April 2023	IW	41.7–46.1	Ascending	13 × 14
07 May 2023	IW	40.1–42.1	Descending	13 × 14
09 May 2023	IW	42.3–44.5	Ascending	13 × 14
19 May 2023	IW	38.1–41.7	Descending	13 × 14

**Table 2:** The mean ± standard deviation values of $C_{11}$, $C_{12}^{i}$, $C_{12}^{r}$, and $C_{22}$ for different soil moisture percentages. Here, *i* stands for the imaginary and *r* stands for the real part of a complex number.

Soil Moisture Range	$C_{11}$	$C_{12}^{i}$	$C_{12}^{r}$	$C_{22}$
0–4.9%	0.030043 ± 0.017466	−0.000262 ± 0.002927	−0.000390 ± 0.002677	0.007928 ± 0.006652
5–9.9%	0.038554 ± 0.016041	0.000319 ± 0.003465	−0.000109 ± 0.003037	0.009798 ± 0.004416
10–19.9%	0.038862 ± 0.015381	−0.000179 ± 0.002856	−0.000140 ± 0.003620	0.009453 ± 0.003810
20–30%	0.039420 ± 0.017366	−0.000501 ± 0.003349	−0.001172 ± 0.002923	0.009023 ± 0.004134

**Table 3:** Comparison of Oh model (Oh et al. [8]), proposed modified Oh model (Mod. Oh), and Chang model (Chang et al. [56]) based on metrics.

Land-Use	Model	Evaluation Metrics				
		RMSE	MAE	MAPE	$\chi_{\nu }^{2}$	*p*
Brinjal	Oh et al. [8]	0.134	0.12	0.93	0.18	<0.001
	Mod. Oh	0.100	0.08	0.48	0.36	0.001
	Chang et al. [56]	0.118	0.10	0.73	0.24	0.02
Fallow	Oh et al. [8]	0.158	0.12	4.12	0.97	<0.001
	Mod. Oh	0.108	0.08	2.22	1.1	<0.001
	Chang et al. [56]	0.120	0.11	3.41	0.41	0.81
Weed	Oh et al. [8]	0.099	0.06	2.64	0.59	<0.001
	Mod. Oh	0.059	0.04	1.21	0.79	0.005
	Chang et al. [56]	0.066	0.05	1.89	0.35	0.001
Sesame	Oh et al. [8]	0.193	0.16	2.69	0.71	<0.001
	Mod. Oh	0.093	0.07	0.80	0.85	0.007
	Chang et al. [56]	0.112	0.09	1.78	0.25	<0.001
All points	Oh et al. [8]	0.154	0.12	3.40	0.84	0.05
	Mod Oh	0.098	0.07	1.70	1.03	<0.001
	Chang et al. [56]	0.108	0.09	2.42	0.37	0.06

RMSE: Root Mean Square Error, MAE: Mean Absolute Error, MAPE: Mean Absolute Percentage Error, $\chi_{\nu }^{2}$: Reduced Chi-Squared, *p*: *p*-value.

## Data Availability

The Sentinel-1 SLC data were accessed from https://search.asf.alaska.edu/#/ on 19 November 2024. The details of the open access data are mentioned in Table 1.
